# Increasing primary and specialty healthcare access for rural older adults: Facilitators, barriers, and opportunities of telehealth in rural community centers

**DOI:** 10.1002/alz.095045

**Published:** 2025-01-09

**Authors:** Ben Fischer, Yolanda Jackson, Celeste Roberts, Celeste Robinson, Robert Early, Robert Sprang, Elizabeth K. Rhodus

**Affiliations:** ^1^ University of Kentucky, Lexington, KY USA; ^2^ Bluegrass Area Agency on Aging and Independent Living, Lexington, KY USA; ^3^ Sanders‐Brown Center on Aging, Lexington, KY USA

## Abstract

**Background:**

Shortcomings in primary and specialty healthcare access in rural communities create substantial health disparities among older adults, especially in areas of modifiable risk factors for Alzheimer’s disease and related dementias (ADRD). Telehealth stations in community centers increase healthcare access to patients thereby improving modifiable risk factors for ADRD. The objective of this project was to assess facilitators, barriers, and opportunities of telehealth use for older adults within rural community centers.

**Methods:**

Following an implementation‐based clinical trial (NCT05552638), this study conducted in‐depth interviews with multi‐level stakeholders, including rural, community‐residing seniors, senior center administrators, and medical partners to assess use of telehealth in rural community centers. Data were analyzed using descriptive thematic analysis. Coding was conducted using HyperResearch Software.

**Results:**

Interviews were completed with nine multi‐level stakeholders who represented of a combine population of >2000 older adults residing in rural communities. Emergent themes reveal facilitators of involvement of community centers as a strategy to promote telehealth usage, as well as involving local providers; both offering familiarity to local residents. Identified barriers include a need for increased acceptance of telehealth services possibly through education and exposure to the technology, patients’ concerns regarding costs, quality of care delivered, and patients’ need for assistance to manage scheduling. Overall, increasing acceptance and fostering positive experiences with technology provide opportunities of improving primary and specialty care via telehealth usage in rural community centers.

**Conclusion:**

Facilitators, barriers, and opportunities for primary and specialty healthcare via telehealth among older adults in rural communities provides key considerations when developing approaches to address health disparities. Findings of this study provide future implementation strategies to improve acceptance and utilization of telehealth services. Continued research is needed to explore health beliefs of healthcare access mechanisms, as well as innovations to address critical health disparities in rural communities.

